# The SCHWIND AMARIS Total-Tech Laser as An All-Rounder in Refractive Surgery

**DOI:** 10.4103/0974-9233.48868

**Published:** 2009

**Authors:** Maria Clara Arbelaez, Samuel Arba Mosquera

**Affiliations:** 1 From Muscat Eye Laser Center, Muscat, Sultanate of Oman; 2 From Grupo de Investigaclün de Cirugía Refractiva y Calidad de Visiön, Instituto de Oftalmobiologia Aplicada, University of Valladolid, Valladolid, Spain; 3 From SCHWIND eye-tech-solutions, Kleinostheim, Germany

**Keywords:** LASIK, Customized Treatments, Aspheric, Aberrations, Wavefront

## Abstract

**Purpose:**

To describe and argument an overview of the main features and unique technical points of AMARIS Total-Tech Laser, coupled with patient outcomes supporting the decision to perform LASIK treatments with maximised outcomes.

**Settings:**

Dr. M.C. Arbelaez, Muscat Eye Laser Center, Muscat, Sultanate of Oman.

**Methods:**

The findings collected during 18-month experience using SCHWIND AMARIS Total-Tech Laser have been reviewed to provide arguments for supporting the decision to perform LASIK treatments with maximised outcomes. For updated clinical outcomes, the last 100 myopic astigmatism treatments, the last 100 hyperopic astigmatism treatments, the last 30 ocular-wavefront-guided treatments, and the last 30 corneal-wavefront-guided treatments, all with 6-month follow-up, were included. For all those, LDV femtosecond system was used to prepare the flaps, and AMARIS flying spot system was used to perform ablations. Clinical outcomes were evaluated in terms of predictability, refractive outcome, safety, wavefront aberration, and contrast sensitivity.

**Results:**

6-month postoperatively, mean defocus was −0.14±0.31D and astigmatism 0.25±0.37D. 70± eyes were within ±0.25D of emmetropia. 43± eyes gained lines of best spectacle-corrected visual acuity. For Aberration-Free treatments, none of the aberration metrics changed from pre- to postoperative values in a clinically relevant amount. For ocular-wavefront-guided treatments, the surgery did not change coma or spherical aberration, and reduced trefoil (p<0.005). For corneal-wavefront-guided treatments, the trefoil, coma, and spherical aberrations, as well as the total root-mean-square values of higher order aberration, were significantly reduced (p<.05) when the pre-existing aberrations were greater than the repeatability and the biological noise.

**Conclusions:**

Although this review does not allow for evidence-based conclusions, following our strategy, LASIK results were excellent. LASIK surgery with AMARIS system yield excellent outcomes. Refractions were reduced to subclinical values with no induction of High-Order-Aberrations. Neither adverse events nor complications were observed.

Aberrations are alterations of the optical surfaces of the eye that lead to deviations in the light entering the eye,^1^ causing a decline in visual quality and a loss of contrast sensitivity.^2^ These aberrations are essentially due to two structures, the cornea and the lens. The remaining structures can also contribute to aberrations in forms such as vitreous condensation, or even tearfilm, but to a lesser degree. Aberrations can be divided into two groups, the low order, which is best known as the spherical (myopia and hyperopia) and astigmatic defects, and a second group of high-order aberrations. Among the high-order aberrations, the spherical and comatic aberrations have the greatest importance.^3^

Aberrations change with age. A clear example of that occurs for the spherical aberration (SA). In a cornea of a young subject SA is positive, and this remains positive with age, however SA of the crystalline lens in a young subject is negative and suffers positivization with age, therefore, in a young subject compensation of the SA occurs, obtaining a total SA which tends to zero, while the total SA with age tends to be positive.

As of today, it is clearly demonstrated that the treatments for the correction of the ametropias with the use of excimer laser induce corneal aberrations. Many studies have proven this.^4^–^10^ Aberrations, whether one's own eye or induced by the treatments, are responsible for poor quality and loss of visual contrast sensitivity. It is for this reason that all manufacturers of laser devices for refractive surgery have developed commercial platforms for the non-induction of aberrations in their treatments, or even to correct the aberrations preexisting in individual eyes.

It is not yet proven that the best is the eye free of aberrations. Some hypotheses state that there are “good” aberrations and others that are to be avoided.^11^–^15^

For correcting aberrations we can focus on two aspects, the correction of corneal aberrations exclusively, or the correction of total eye aberrations.

Excimer laser refractive surgery has evolved from simple myopic ablations^16^ to the most sophisticated topography-guided^17^ and wavefront-driven,^18^ either using wavefront measurements of the whole eye (obtained, e.g., by Hartman-Shack wavefront sensors) or by using corneal topography-derived wavefront analyses^19^,^20^ customised ablation patterns. Because the corneal ablations for refractive surgery treatments induce aberrations (one of the most significant side-effects in myopic LASIK is the induction of spherical aberration,^21^ which causes halos and reduced contrast sensitivity), special ablation patterns were designed to preserve the preoperative level of high order aberrations.^22^–^24^ Patient satisfaction in any refractive surgery, wavefront-guided or not, is primarily dependent on successful treatment of the lower order aberrations (LOA) of the eye (sphere and cylinder). Achieving accurate clinical outcomes and reducing the likelihood of a retreatment procedure are major goals of refractive surgery.

Wavefront, aspheric and conventional laser in situ keratomileusis (LASIK) for the treatment of myopia and myopic astigmatism is safe and effective.^25^,^26^ The recent advances in excimer laser technology, such as the use of aspheric ablation profiles, incorporation of higher order aberration (HOA) treatment and eye trackers have presumably led to better refractive outcomes and reduced HOA induction postoperatively that have been recently reported.^27^,^28^ Although most laser manufacturers incorporate the suite of products mentioned above,^25^ the use of high repetition rates (500 Hz or higher) to reduce treatment times have not been widely adopted. This is likely due to technological constraints and the increased thermal effect concomitant with the use of higher repetition rates.^29^,^30^ If left unaddressed, the thermal effect can cause tissue damage^29^,^30^ and potentially reduce refractive outcomes.^31^ The reduction of treatment times may be beneficial due to: 1. shorter treatment times that may result in less stromal dehydration; and 2. patients are less likely to lose fixation during the ablation.^32^,^33^

Reasonable reductions in HOAs after wavefront-guided treatments on aberrated eyes and reasonable changes in HOAs after wavefront-optimized treatments have been reported.^34^,^35^ However, ocular wavefront-guided and conventional treatments can increase HOAs by 100% postoperatively.^27^ A significant number of refractive surgery patients may not benefit from ocular wavefront guided treatment as the induction of HOAs is related to baseline levels of HOAs.^27^,^36^ For example HOAs tend to be induced in patients with less than 0.30 μm and reduced in patients with greater than 0.30 μm of HOAs.^27^,^36^ Physiologic optical aberrations may be required to maintain the optical quality of the eye.^37^,^38^ Based on these studies,^27^,^36^,^37^,^38^ it seems that customised ablation algorithms in any form (ocular wavefront guided, corneal wavefront guided, topography guided, etc.) may not be appropriate for the entire refractive surgery population (i.e. specific population groups have specific demands, and deserve specific treatment solutions. No one-size-fits-all concept can be applied).

Our definition of “Customisation” is conceptually different and can be stated as: “The planning of the most optimum ablation pattern specifically for each individual eye based on its diagnosis, and visual demands”. It is often the case, that the best approach for planning an ablation is a sophisticated pattern, which can still be simply described in terms of sphere, cylinder, and orientation (axis).

## PATIENTS AND METHODS

A retrospective study in which we studied 360 eyes operated using the technique of LASIK with the AMARIS laser (SCHWIND eye-tech-solutions GmbH & Co.KG, Mainparkstrasse 6-10, Kleinostheim, Germany) is presented here. 4 study groups were conformed: 100 eyes manifesting preoperative myopia with astigmatism, 100 eyes treated for hyperopia with astigmatism, 30 eyes treated by Corneal Wavefront, and 30 eyes treated by Ocular Wavefront.

In all eyes, we measured corneal topography^39^ and derived corneal wavefront^19^ analyses (Keratron-Scout, OPTIKON2000, Rome, Italy), ocular aberrometry and derived ocular wavefront analyses (Ocular Wavefront Analyzer, SCHWIND eye-tech-solutions GmbH & Co.KG), manifest refraction, and uncorrected and best spectacle-corrected Snellen visual acuity^40^ (UCVA and BSCVA respectively).

A specific informed consent for the study was not required because of its retrospective nature and the fact that we did not perform any action on the patient other than the usual LASIK procedure.

All ablations were calculated using the ORK-CAM software module. Aspheric aberration neutral (Aberration-Free™) profiles for the baseline are not based on the Munnerlyn proposed profiles,^16^ and go beyond that by adding some aspheric characteristics to balance the induction of spherical aberration^41^,^42^ (prolateness optimization^43^,^44^). The aberration neutral (Aberration-Free™) profile is aspherical-based,^45^ including a multidynamic aspherical transition zone, aberration and focus shift compensation due to tissue removal, pseudo-matrix based spot positioning, enhanced compensation for the loss of efficiency,^46^ and intelligent thermal effect control; all based on theoretical equations validated with ablation models and clinical evaluations.

A 6.5 mm central fully corrected ablation zone was used in all eyes with a variable transition size automatically provided by the laser related to the planned refractive correction (6.7 mm to 8.6 mm). The AMARIS excimer laser is a flying-spot laser using real ablative spot shape (volume) locally considered through a self-constructing algorithm. In addition, there are a randomized flying-spot ablation pattern and controls for the local repetition rates to minimize the thermal load of the treatment.^30^ Therefore, the ablated surface in the aspheric aberration neutral (Aberration-Free™) profiles should be very smooth, so that there will be some benefits in high order aberrations. Finally, all these optimizations theoretically diminish the induced wavefront aberration after myopic LASIK. This system works at a true repetition rate of 500 Hz and produces a beam size of 0.54 mm FWHM with a superGaussian ablative spot profile.^47^,^48^ High-speed eye-tracking (pupil and limbus tracker with cyclotorsional tracking) with a 1050-Hz acquisition rate is accomplished with a 3-ms latency time^49^.

All flaps were created using a FEMTO LDV™ femtosecond laser (Ziemer Group, Port, Switzerland) with 110 μm nominal flap thickness.

Optical errors centred on the line-of-sight, representing the Wavefront Aberration, are described by Zernike polynomials^50^ and coefficients in OSA standard,^51^ and analysed for a standardised diameter of 6 mm for corneal wavefront.

**Figure 1:**
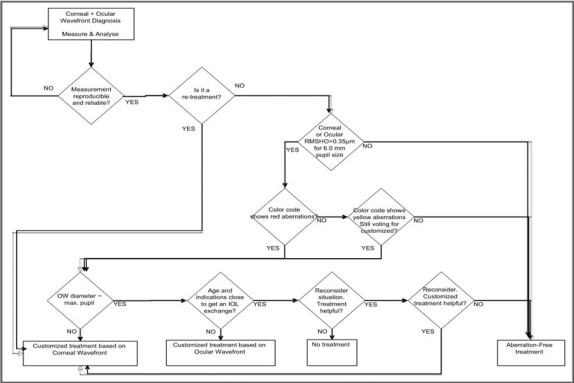
Decission-Tree applied for selecting the treatment mode (Aberration-Free, Corneal WavefrontGuided, or Ocular Wavefront Guided)

For selecting the type of correction to be applied (Aberration-Free, Corneal Wavefront Guided or Ocular Wavefront Guided), the Decission-Tree depicted in Fig. 1 was applied.

Finally we reported, as well, the retreatment rate for each subgroup considering the total number of treatments performed since we started using the SCHWIND AMARIS.

## STATISTICAL ANALYSIS

Comparative statistical analyses were conducted using Student's t-tests based on the changes in the corneal wavefront aberrations induced by refractive surgery in all subgroups of the population studied, comparing the postoperative wavefront aberrations to the preoperative baseline. We assessed whether the changes were statistically significant within the groups (Student's t-tests for paired data). The level of statistical significance was taken as p<.05.

## RESULTS

Six-month postoperatively, mean defocus was −0.14±0.31D and astigmatism 0.25±0.37D (very similar for all subgroups).

**Figure 2:**
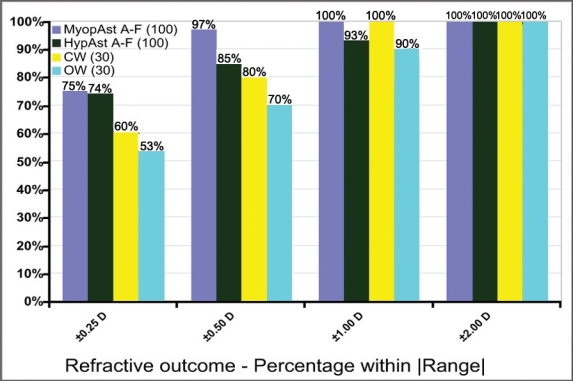
Refractive outcome

75% eyes were within ±0.25D of emmetropia, and 100% within ±1.00D in the Myopic-Astigmatism Aberration-Free subgroup; 74% eyes were within ±0.25D of emmetropia, and 93% within ±1.00D in the Hyperopic-Astigmatism Aberration-Free subgroup; 60% eyes were within ±0.25D of emmetropia, and 100% within ±1.00D in the Corneal Wavefront subgroup; and 53% eyes were within ±0.25D of emmetropia, and 90% within ±1.00D in the Ocular Wavefront subgroup (Fig. 2).

**Figure 3:**
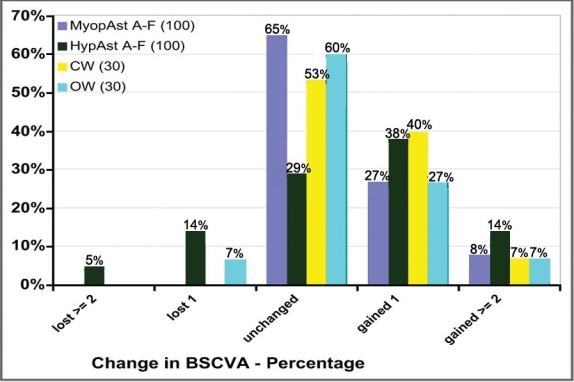
Safety

35% eyes gained 1 or more lines of best spectacle-corrected visual acuity, and no single eye lost more than 1 line of BSCVA in the Myopic-Astigmatism Aberration-Free subgroup; 52% eyes gained 1 or more lines of best spectacle-corrected visual acuity, and only 5% eyes lost more than 1 line of BSCVA in the Hyperopic-Astigmatism Aberration-Free subgroup; 47% eyes gained 1 or more lines of best spectacle-corrected visual acuity, and no single eye lost more than 1 line of BSCVA in the Corneal Wavefront subgroup; and 33% eyes gained 1 or more lines of best spectacle-corrected visual acuity, and no single eye lost more than 1 line of BSCVA in the Ocular Wavefront subgroup (Fig. 3).

**Figure 4:**
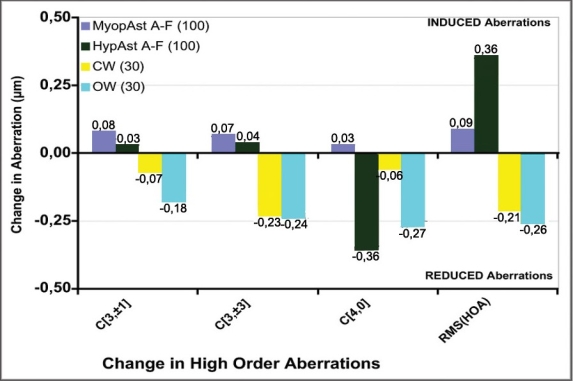
Comparison of induced aberrations at 6-mm analysis diameter

For Aberration-Free treatments, none of the aberration metrics changed from pre- to postoperative values in a clinically relevant amount. For ocular-wavefront-guided treatments, the surgery did not change coma or spherical aberration, and reduced trefoil (p<0.005). For corneal-wavefront-guided treatments, the trefoil, coma, and spherical aberrations, as well as the total root-mean-square values of higher order aberration, were significantly reduced (p<.05) when the pre-existing aberrations were greater than the repeatability and the biological noise (Fig. 4).

The rate of aberration induction per dioptre of achieved defocus correction was 0.02 μm/D for the myopic baseline, and 0.05 μm/D for the hyperopic baseline.

Overall, in the 18 months we are using the SCHWIND AMARIS, we have performed 3880 LASIK treatments divided as: 3157 treatments for myopic-astigmatism using Aberration-Free profiles, 67 treatments for hyperopic-astigmatism using Aberration-Free profiles, 336 treatments using Corneal Wavefront Guided profiles, and 320 treatments using Ocular Wavefront Guided profiles. From those, we have performed 13 retreatments overall (0.3%): 10 retreatments (0.3%) after myopic-astigmatism using Aberration-Free profiles, no retreatments (0.0%) after hyperopic-astigmatism using Aberration-Free profiles, 2 retreatments (0.6%) after Corneal Wavefront Guided profiles, and 1 retreatment (0.3%) after Ocular Wavefront Guided profiles.

## DISCUSSION

It was already known time ago that the treatment of ammetropias with excimer laser induced aberrations.^4^–^10^ Since then, laser platforms have made changes in their systems to minimize the induction of these^22^–^24^ and some have even tried to correct all the aberrations,^17^,^18^ although there are studies showing that a certain number of aberrations, or a specific combination of them can provide better vision.^11^–^14^ There are three types of approaches. The first are those that have as their objective the elimination or reduction of the total aberrations of the eye.^18^ The main critics of this approach have argued that aberrations (especially the ones of the crystalline lens) change significantly with age. In addition there are changes in the aberrations (especially the ones of the crystalline lens) during accommodation, so the goal Ñzero aberration“ would be inconsistent throughout the day due to accommodation, and little lasting, since aberrations change with age.^52^–^54^ Not to mention the changes in aberration due to the tear film. The second approach is intended to correct the corneal aberrations,^17^ and we know that the corneal aberrations do not change with age.^55^,^56^ However this concept might also be wrong considering corneal aberrations interact with internal aberrations, some of them being cancelled, and producing an aberration pattern of the total eye in general different from the aberration pattern of the cornea alone.^20^ So by only removing corneal aberration we might worsen the overall aberrations, since the internal aberration in this case will not find a corneal aberration for compensation. In case that the corneal aberration is of the same sign as the internal aberration, the correction of the corneal aberration would be very useful as it would reduce the aberration of the total eye. This is why it can not be given any of the two types of treatments described previously indiscriminately to everyone. Both can be useful, but require a prior study of corneal and internal aberrations (in a non-accommodated state), to know whether and which aberrations are balanced and which ones are not, and whether a particular type of aberration is better to be removed or to leave it like it is. A third and last type of approach tries not to induce aberrations due to treatment with the excimer laser. What is intended is to leave the eye in terms of high-order aberrations as it was before the treatment. This type of treatment is not as ambitious, but much more simple to operate and can be applied to all patients in an indiscriminate way and without any prior study. The aim is by means of inducing no aberrations, to achieve an improved postoperative visual quality. The advantage of the Aberration-Free™ ablation profile is that it aims being neutral for HOA, leaving the visual print of the patient as it was preoperatively with the best spectacle correction.

Our follow-up included 100% of the cases at 6 months and, despite no nomogram adjustments were applied, the retreatment rate was below 0.3%. As a proof of stability, longer follow-up and larger number of eyes would be more convincing, even though, refractive spherical and astigmatic results are stable after 3 months.

Comparing myopic and hyperopic groups, statistical significance was observed with a less spherical aberration induction in myopia compared to hyperopia only for AMARIS in all refraction subgroups (unpaired t-tests, p<.0005). Similar findings were reported by Llorente et al.^57^ They measured induced corneal HOA's after conventional treatments. For 6mm analysis diameter they found a rate of induced spherical aberration of 0.17 μm/D for myopia and 0.28 μm/D for hyperopia. Kohnen et al.^8^ measured induced corneal HOA's after conventional treatments. For 6mm analysis diameter they found a rate of induced spherical aberration of 0.04 μm/D for myopia and 0.07 μm/D for hyperopia.

The main high order aberration effects post-op (coma and spherical aberration) are coming from decentration and “edge” effects, the strong local curvature change from Optical Zone to Transition Zone and from Transition Zone to non-treated cornea. Then it is necessary to emphasize the use of huge Optical Zones, covering the scotopic pupil size plus some tolerance for possible decentrations, and well-defined smooth Transition Zones.

Long-term follow-up on these eyes will help determine whether these accurate results also show improved stability compared to previous experiences.

The present investigation of LASIK using the SCHWIND AMARIS excimer laser with a 500 Hz repetition rate found this laser platform is safe, predictable and gives stable results. For example, 70 % of eyes were within 0.25 D of intended correction (Fig. 2). Safety was demonstrated with only 1% eyes losing more than 1 line of BSCVA 6 months after surgery (Fig. 3).

There is evidence of neural adaption to the baseline wavefront profile.^38^ The interaction between higher order aberrations can be beneficial to visual quality regardless of the magnitude HOAs.^13^,^11^ To date the induction of wavefront aberrations postoperatively is random and the wavefront profile postoperatively cannot be predicted. Based on the random nature of the HOA induction and current research,^36^–^38^ it maybe beneficial to maintain the preoperative wavefront profile for a significant number of refractive surgery candidates.

The changes in spherical aberration can be partially explained by the biomechanical response and corneal epithelial remodeling.^58^,^59^ The mild induction of HOAs, spherical aberration and coma may explain the maintenance of visual quality postoperatively found in the current study.

Based on the results presented here, we are however not postulating that customised ablation algorithms in any form (ocular wavefront guided, corneal wavefront guided, topography guided, etc.) are not be useful. Rather, than specific populations with specific demands deserve specific treatment solutions. Aspheric treatments aiming for preservation of the preoperative HOAs show their strengths in patients with preoperative BCVA 20/20 or better, or in patients where the visual degradation cannot be attributable to the presence of clinically relevant HOAs.

The use of a 500 Hz repetition rate did not create any postoperative complications. Thermal effects due to excimer laser ablation and associated plume debris have been previously reported.^29^,^30^,^60^,^61^ One study reported better refractive outcomes after cooling the cornea prior to the ablation which likely reduced the thermal buildup.^31^ The use a particle aspirator coupled with random placement of the laser spots in this study seems to efficiently speed up the treatment without increasing the thermal load in the cornea.

The 6 month results presented in this study indicate that Aberration-Free™ treatments of myopia with and without astigmatism using the SCHWIND AMARIS is safe, gives excellent refractive and visual acuity results, and maintains preoperative contrast sensitivity.

Corneal wavefront guided treatments showed promising results in this study. Based on the findings 6 months postoperatively, it could be concluded that the

CW customized treatments AMARIS produced both safe and predictable ablation of the cornea. The CW group showed an average change in coma from 0.38 μm to 0.31 μm (−19%) (P = 0.04), in trefoil from 0.35 μm to 0.12 μm (−66%) (P = 0.0005), and in spherical aberration from +0.14 μm to +0.08 μm (−48%) (P = 0.02). The accuracy, predictability, and stability of the refractive power change, together with the minimal external impact of the AMARIS ablation profiles on the HOAs, led to very good results in terms of visual quality.

The CW customized approach shows its strength in cases where abnormal corneal surfaces are expected. Apart from the risk of minimal additional ablation of corneal tissue, systematical wavefront-customized corneal ablation can be considered as a safe and beneficial method.

In the OW group, all ablations were customised based on Hartmann-Shack measurements of the wavefront aberration of the entire eye and calculated using the ORK-CAM software module. The ORK-CAM software module is able to import, visualize, and combine diagnostic data of the eye (manifest refraction and ocular wavefront data in this case) into a customised aspherical ablation profile to optimize the corneal shape. As we used OW based profiles in this group, ablations were optimized to reduce the wavefront aberration of the entire eye (within Optical Zone, OZ) close to a zero level, compensating, as well, for the aberration induction observed with other types of profiles.

The improvement in safety was statistically significant (*P* = 0.04) with 33% of the eyes treated improving BSCVA. 7% of the eyes “have lost” 1 line of BSCVA in the OW group, however no single eye has lost more than 1 line of BSCVA. The repeatability of the BSCVA within individuals from day to day is about 1 line of BSCVA. Since only 2 eyes lost 1 line of BSCVA, we have reviewed previous follow-ups of those eyes, and observed that in one eye this loss was present at all follow-up times, whereas the other eye showed the above mentioned variability, i.e. no loss at 3-month follow-up compared to baseline.

The correction of trefoil terms was successful both in magnitude (*P =* 0.002) and correlation attempted versus achieved (*P* < 0.0001), whereas the decrease in spherical aberration was not statistically significant (*P* = 0.05), but correlation attempted versus achieved was successful (*P* < 0.0001). It should be noted that opposing the preoperative wavefront aberration in laser refractive surgery constituted only a first approximation of a perfect refractive correction, as tissue removal occurs.

Our data suggest that wavefront customized treatments can only be successful, if the pre-existing aberrations are greater than the repeatability and the biological noise. Furthermore, coupling effects between different high order aberration terms, and between HOAs and manifest refraction is still one of the major sources of residual aberrations after refractive surgery. This topic has been discussed from a theoretical perspective by Baró et al.^62^ and from a clinical perspective by MacRae^63^ or Buehren et al.^64^ They all found mutually affecting interactions, for example, between defocus and spherical aberration, or between 3 order aberrations and low order terms, between spherical aberration and coma, or between secondary and primary astigmatisms.

Both wavefront-guided groups corroborate other findings that have been recently published. The fact that in order to appropriately treat patients with profiles such as these described the patients need to have a significant level of preoperative aberrations. Recently published independent studies by Stonecipher et al.^34^ and by Venter^65^ illustrated similar findings.

Our study demonstrated that aspheric ablation profiles, designed with CAM software for the AMARIS laser platform, are safe and yielded visual, optical, and refractive results comparable to those of other wavefront-guided customized techniques for correction of myopia and myopic astigmatism.^18^,^23^,^34^,^65^ In particular, the wavefront guided approaches are highly efficient in eyes with greater than 0.35 microns RMS HOA, or where individual components of the wavefront aberration such as coma, trefoil or spherical aberration are greater than 0.25 microns RMS.

From the analysis presented here, can be concluded that the Wavefront-guided treatments are not always the Gold Standard.

Wavefront customised treatments (both Corneal or Ocular Wavefront) can only be reasonably successful when the pre-existing aberrations are huger than the repeatability, biological noise and accommodation effects. Considerations as treatment duration or tissue removal make more difficult to establish a universal optimal profile.

General optimum in non-wavefront-driven refractive surgery is to balance the effects on the Wavefront aberration, and, to provide normal eyes with the best quality of vision, without affecting their perception of the world:

Ocular Wavefront treatments have the advantage of being based on Objective Refraction of the complete human eye system; Corneal Wavefront treatments have the advantage of being independent from accommodation effects or light/pupil conditions; Aspherical treatments have the advantage of saving tissue, time and a due to their simplicity they offer a better predictability.
